# Enhancement of γ-Aminobutyric Acid and the Characteristics of Nutrition and Function in White Quinoa through Ultrasound Stress at the Pre-Germination Stage

**DOI:** 10.3390/foods13010057

**Published:** 2023-12-22

**Authors:** Mengying Wu, Qian Zhou, Liangfu Zhou, Jie Wang, Ting Ren, Yu Zheng, Wei Lv, Wen Zhao

**Affiliations:** 1 College of Food Science and Technology, Agricultural University of Hebei, Baoding 071001, China; wumy9902@163.com (M.W.); zhouqian@hebau.edu.cn (Q.Z.); zhoulf202201@163.com (L.Z.); wangjie@hebau.edu.cn (J.W.); rt1134771972@163.com (T.R.); zhengyu9818@163.com (Y.Z.); 2 National Engineering Research Center for Semi-Arid Agriculture, Shijiazhuang 050000, China; lvwei91@126.com

**Keywords:** *Chenopodium quinoa* Willd., abiotic stress, γ-aminobutyric acid, functional properties, nutritional properties

## Abstract

The global production of quinoa has been increasing in recent years. In plant-based foods, ultrasound stress has received increasing attention, owing to its ability to enhance the production of primary and secondary metabolites. We studied the effects of ultrasonic stress at the pre-germination stage on the γ-aminobutyric acid (GABA) accumulation and characteristics of nutrition and function in quinoa. The results showed that ultrasonic conditions of 100 W for 4 min promoted an increase in GABA content by 9.15-fold, to 162.47 ± 6.69 mg/100 g·DW, compared to that of untreated quinoa, through promoting a 10.2% and 71.9% increase in the water absorption and glutamate decarboxylase activity of quinoa, respectively. Meanwhile, compared to untreated quinoa, ultrasonic stress at the pre-germination stage enhanced the total phenolic, total flavonoid, and total saponin contents of quinoa by 10.2%, 33.6%, and 90.7%, to 3.29 mg GA/g·DW, 104.0 mg RE/100 g·DW, and 7.13 mg/g, respectively, without decreasing its basic nutritional quality. Ultrasonic stress caused fissures on the surface of quinoa starch particles. Additionally, germination under ultrasonic stress increased the n3 polyunsaturated fatty acids by 14.4%. Furthermore, ultrasonic stress at the pre-germination stage promoted the scavenging of 2,2-diphenyl1-picrylhydrazyl radicals and inhibitions of α-amylase, α-glucosidase, and pancreatic lipase by 14.4%, 14.9%, 24.6%, and 20.0% in vitro, compared to untreated quinoa. The results indicated that the quinoa sprouted via ultrasonic stress could represent a promising method through which to develop nutritionally balanced whole grains rich in GABA, with hypoglycemic and hypolipidemic activities, which could provide theoretical support for the development of functional whole-grain foods based on quinoa.

## 1. Introduction

Quinoa (*Chenopodium quinoa* Willd.) is an annual herbaceous plant. This food benefits gluten-sensitive individuals, owing to its gluten-free nature [1]. The quinoa plant is native to the Andes of South America and dates from 5000 BC to 3000 BC; it was introduced to China in the 1980s and cultivated on a large scale after 2012. Quinoa is mainly cultivated in Shanxi, Gansu, Inner Mongolia, Qinghai, Tibet, Hebei, and other regions in China. The FAO declared 2013 as the International Year of Quinoa. In 2022, the global quinoa market amounted to approximately USD 91.73 billion, and it is estimated to exceed USD 152.44 billion by 2027. Moreover, global quinoa production has increased, from 80,000 metric tons in 2010, to 147,000 metric tons in 2021, as per an FAO statement [2]. Quinoa is a pseudo-cereal that is abundant in carbohydrates (67–74%), proteins (10–18%), lipids (4.4–8.8%), and phytochemicals such as polyphenols. Some studies have shown that the presence of amylose in quinoa ranged from 7% to 27%, and that the sizes of its starch granules were lower than those of corn (1–2 μm) or wheat (2–40 μm) [3]. Quinoa proteins constitute all essential amino acids, with lysine and leucine contents equivalent to 96% and 91% of the FAO standards, respectively [3]. The quinoa seed is a major source of essential fatty acids, with the main fatty acid fractions being linoleic acid (18:2n-6), linolenic acid (20:3n-6) (55–60%), and oleic acid (18:1 *cis*-9) (30%) [3]. Additionally, the polyphenols in quinoa grains are dominated by free phenols (ranging from 167.2 to 308.3 mg gallic acid equivalents per 100 g dry weight), which play important roles in the prevention of both diabetes and obesity [4,5]. Meanwhile, studies have shown that quinoa reduces serum triglyceride levels and metabolic syndrome [6,7].

Germination, as a biochemical process, can enhance the nutrients in whole grains. Germination of different grains is triggered by specific conditions, including optimal temperature, light, and moisture to support plant growth. Suitable germination conditions activate the relevant enzymes and hormones, transforming the grain from dormancy to active metabolism and facilitating the release of nutrients [8]. Products based on germinated grains represent new components in the food industry that have enhanced nutritional value, mineral absorption, flavor, and taste [9]. Sprouted grain products, such as sprouted mung beans and sprouted brown rice, have become very common in the market [10]. Germination has a positive effect on the bioavailability of severely limited phenolic acids in grains [11]. Previous results have shown that the γ-aminobutyric acid (GABA) levels (22.41 mg/100 g·DW), TPC (270.99 mg GAE/100 g·DW), and oxygen radical absorbance capacity (1085.75 mg TE/100 g·DW) of ungerminated quinoa could be increased to 122.32 mg/100 g·DW, 499.24 mg GAE/100 g·DW, and 1437.19 mg TE/100 g·DW, respectively, through germination [12]. Interestingly, the opposite results were observed for saponins. One study indicated that the most abundant saponins showed decreases in their contents during normal germination and increased markedly during germination under selenium stress [13]. Another study demonstrated that sprouted quinoa showed a higher total saponin content (TSC) [14].

GABA is an essential non-protein amino acid. The various health benefits of GABA-enriched foods mainly include neuroprotection, anti-depression, anti-insomnia, anti-diabetes, anti-hypertension, and anti-inflammation properties [15]. Many studies have shown that GABA accumulates in large quantities when plants are subjected to abiotic stresses, and acts as a signaling molecule to regulate tolerance to various abiotic stresses [16]. Common pre-germination abiotic stress techniques include low-temperature, salt, hypoxic, and ultrasound stress [15]. In plant-based foods, ultrasound stress has received increasing attention as a burgeoning non-thermal technology, owing to its ability to enhance the production of primary and secondary metabolites [17]. For germinated wheat and red rice, samples treated with ultrasound (25 kHz for 5 min) accumulated various metabolites, including beneficial (such as riboflavin and GABA) and flavorful (such as glucose and free sugar) plant metabolites [18]. Meanwhile, germinated oats stimulated with ultrasound for 5 min after soaking showed high levels of GABA, succinic acid, alanine, total avenanthramides, and TPC at different germination times [19]. A study on whole-grain brown rice indicated that ultrasound treatment has stimulatory effects on starch decomposition; increases the reducing sugar, GABA, proline, and antioxidant contents; and simultaneously improves the nutritional index of free amino acids and the bioaccessibility of iron and calcium in vitro [20]. The influence of ultrasound-assisted sprouting on bioactive phytochemicals was considered to be related to stress effects [18], ultimately affecting plant phenotypes. In the early germination stage of the common bean, increased auxin levels in the radicle tip in response to ultrasonic stimulation may positively affect root growth with increasing treatment intensity [21]. However, the average root length was reduced by 20.41% at 300 W, relative to that of the matched control group [22]. Common pre-germination abiotic stresses, such as low temperatures, salt, and hypoxia, may cause harmful effects on plant growth [15], which can be avoided or even promoted under appropriate sonication conditions.

Although GABA is widely found in vertebrates, plants, and microorganisms, it is present in extremely low levels and is unlikely to fulfill human needs. Quinoa seeds are most commonly used for direct cooking, and more highly processed quinoa products are in urgent demand. However, few studies have investigated the effects of germination and ultrasonic stress on the nutritional composition and functional properties of quinoa as a whole-grain dietary supplement. Therefore, we tried to develop a functional whole-grain food with hypolipidemic and hypoglycemic potential, based on quinoa enriched with GABA through ultrasound stress at the pre-germination stage. At the same time, we considered it necessary to determine the effects of ultrasonic stress and germination on the nutritional quality of quinoa. The aim of this study was to investigate the effects of ultrasonic stress at the pre-germination stage on the GABA accumulation, starch, protein, amino acid, and fat properties, as well as on the phytochemicals in quinoa. More importantly, we investigated the effects of ultrasonic stress at the pre-germination stage on the antioxidant, in vitro hypoglycemic, and in vitro hypolipidemic efficacies of quinoa flour. The technical data could provide theoretical support for the intensive processing and development of functional food, based on quinoa, that conforms to the healthy market tendencies and consumer demands for healthy gluten-free and nutritionally balanced whole grains.

## 2. Materials and Methods

### 2.1. Samples and Chemicals

Dehulled quinoa cultivar ‘Jili 3’ was grown and harvested at Zhangjiakou Yuerwan Quinoa Planting Cooperative, which is located in Zhangjiakou City (115°36′ E, 415°27′ N), Hebei Province, China. The seeds were preserved in the dark at −20 °C under vacuum until germination. GABA, with a purity ≥ 99%, and 2,2-diphenyl1-picrylhydrazyl (DPPH) were purchased from Sigma-Aldrich (Shanghai, China). Threonine (99%), valine (99%), methionine (98%), isoleucine (98%), leucine (98%), phenylalanine (98%), lysine (98%), histidine (98%), arginine (98%), aspartate (98%), serine (98%), glutamate (98%), glycine (98%), alanine (98%), cysteine (98%), tyrosine (99%), proline (98%), gallic acid (99%), rutin (95%), saponin (98%), 2,2′-azino-bis (3-ethylbenzthiazoline-6-sulfonic acid) (ABTS) (98%), sodium cholate (65%), sodium taurocholate (98%), sodium deoxycholate (98%), and sodium glycocholate (97%), as well as α-amylase (100,000 U/g), α-glucosidase (50,000 U/g), and pancreatic lipase (30,000 U/g), were obtained from Shanghai Yuanye Bio-Technology Co., Ltd. (Shanghai, China). Acetonitrile (chromatographically pure) was purchased from Macklin Biochemical Co. Ltd. (Shanghai, China). All other chemicals were analytical reagent grade.

### 2.2. Germination of Quinoa

Dehulled quinoa was soaked in NaClO (0.1%) for 30 min for surface sterilization and rinsed with distilled water at least three times until no NaClO residue remained on the surface. After soaking in distilled water at 24 ± 1 °C for 14 h, the quinoa samples were laid in a culture dish with two layers of filter paper and separately placed in a biochemistry cultivation cabinet at 24 ± 1 °C for 24 h. The controlled sprouting process was performed in the dark, and the water was changed every 12 h. After freezing at −20 °C overnight, the samples were vacuum-freeze-dried for 32 h, pulverized, and then kept at −20 °C.

### 2.3. Ultrasonic Treatment

**Figure 1:**
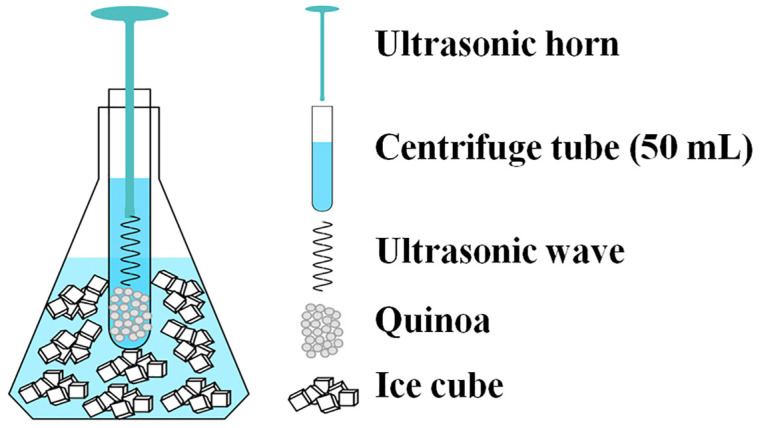
Schematic diagram of ultrasound stress process.

As shown in Figure 1, after surface disinfection, the effects of varying ultrasonic power (0, 50, 100, 150, and 200 W) and time (0, 2, 4, 6, and 8 min) on the GABA content of quinoa sprouts were investigated, using a single-factor test with an ultrasonic cell pulverizer (Scientz-IID, Ningbo Scientz Biotechnology Co., Ltd., Ningbo, China). The ultrasound treatment time and interval time were both 3 s. During the ultrasonic process, an ice water bath was applied to prevent excessive temperature increase. The ultrasonically stimulated quinoa was immediately soaked and sprouted.

### 2.4. Determination of GABA Content, Moisture Absorption, Turbidity of Soaking Water, and Glutamate Decarboxylase (GAD) Activity

The determination of the GABA content was carried out according to a previously reported method, with slight modifications [23]. The samples (1.00 g) were evenly admixed with citric acid (0.01 mol/L, 5.00 g) and shaken for 2 h for GABA extraction. The supernatant was centrifuged at 4000 rpm for 15 min. Then, 1 mL each of the supernatant, NaHCO_3_ (1 mol/L, pH = 9), and 1-fluoro-2,4-dinitrophenylacetonitrile solution (0.1%) were added to a volumetric flask in turn, mixed well, allowed to react at 60 °C for 90 min, and diluted to 10 mL with phosphate buffer (0.01 mol/L, pH = 7.2). After filtration, the GABA content was determined using an Agilent 1260 HPLC with a Universil C18 column (4.6 mm × 250 mm, 5 μm). The mobile phases were phosphate buffer (0.01 mol/L, pH = 7.2): acetonitrile: ultra-pure water = 70:8:22, with a 1-mL/min flow rate. The GABA peak was detected and quantified at 360 nm, at a column temperature of 40 °C.

Moisture absorption was determined based on the difference between the initial mass (3.00 g) and the mass after treatment (the surface water on soaked quinoa grains was wiped with filter papers) divided by the initial mass. The turbidity of the soaking water was determined by transferring quinoa (3.00 g) that had been surface disinfected to a 50 mL centrifuge tube, containing 30 mL of water, for power ultrasound treatment. The absorbance of the soaked water samples was assessed using a spectrophotometer (WFZ UV-2802H, Unico Instrument Co., Ltd., Shanghai, China) at 500 nm. Deionized water was used as a blank [19]. GAD activity was measured according to the instructions of the GAD Elisa Kit (Suzhou Comin Biotechnology Co., Ltd., Suzhou, China).

### 2.5. Analysis of Nutritional Quality

The protein, fat, dietary fiber (DF), and ash contents were analyzed according to Chinese standard methods GB5009.5-2016 [24], GB5009.6-2016 [25], GB5009.88-2014 [26], and GB5009.4-2016 [27]. Starch and reducing sugar analyses were conducted based on a previously described protocol [20], applied with a few modifications. The ground sample was admixed evenly with ethanol (80%, *v*/*v*), and then centrifuged twice (4000 rpm, 15 min), and the precipitate and supernatants were collected separately. HCl (6 mol/L) was used to acid-hydrolyze the precipitate at 100 °C for 20 min. The reducing sugar contents in the supernatants, and the glucose contents in the hydrolysate, which were used for starch content determination, were evaluated according to the 3,5-dinitrosalicylic acid (DNS) colorimetric method. Subsequently, 1 mL of the extract was mixed with 1.5 mL of DNS reagent, reacted in a boiling water bath for 5 min, and cooled to room temperature in an ice water bath. Then, it was fixed to 25 mL with distilled water, and the absorption was measured at 540 nm.

Scanning electron microscopy (SEM) was used to describe the starch morphology and particle size. Starch was extracted and purified as previously described [28]. Dry particles were sprinkled on the double-sided tape connected to the sample stub, a thin layer of gold was applied, and then observations were performed at 40,000-fold magnification using SEM (Tescan Mira4, Brno, Czech Republic) at an acceleration potential of 5 kV.

The proteins in the quinoa and sprouts were extracted using alkali extraction and acid precipitation methods [29]. A solution of flour and distilled water was prepared, in a ratio of 1:10. Protein solutions, whose pH values were adjusted to 8.0 with NaOH (1.0 mol/L), were centrifuged (4000 rpm, 15 min) after stirring (55 °C, 1.5 h) to remove insoluble materials. The supernatants, whose pH values were adjusted to 4.5 with HCl (1.0 mol/L), were stirred for 1.5 h and centrifuged (4000 rpm, 15 min). The precipitates were rinsed with distilled water, the pH was adjusted to neutral, and they were freeze-dried and preserved at −20 °C. The Kjeldahl method was used to determine the protein content. SDS-PAGE was performed on stacking gel (5%) and separating gel (12%), with a thickness of 1 mm, to detect the effect of ultrasound stress on protein patterns [30]. A 10% protein solution (*w*/*v*) was dissolved in 5× sample buffer and boiled for 5 min. Gels (12 wells) were used to load the samples (3 μL), which were subjected to electrophoresis via stacking and separating gels at constant voltages of 80 V and 120 V, respectively. The gels were then stained with Coomassie Brilliant Blue R250. The determination of amino acids was performed using an amino acid analyzer (L 8900, Hitachi, Japan) [31]. The sample (100 mg) was placed in an ampoule, mixed with 10 mL of 6 mol/L HCl, sealed with nitrogen, and hydrolyzed at 110 °C for 24 h. After hydrolysis, the sample was fixed to 50 mL, and 2 mL of the sample was deacidified to dryness at 45 °C on a rotary evaporator. The residual was dissolved in 2 mL of sample buffer and passed through a 0.45 μm membrane filter for on-line detection. The amino acid content was determined using a cationic resin chromatography column (200 mm × 4.6 mm) and an ultraviolet detector. The detection wavelength of proline was 440 nm, and that used for other amino acids was 570 nm, at a column temperature of 55 °C. The chemical score (CS), amino acid score (AAS), and essential amino acid index (EAAI) were calculated based on the amino acid composition [32]. The calculation formulas are as follows:

$$CS=AA_{Fi}/AA_{Ei}\;AAS=AA_{Fi}/AA_{Xi}\;EAAI=\sqrt[{i}]{\left(AA_{F1}/AA_{E1}\right)\times \left(AA_{F2}/AA_{E2}\right)\times \cdots \times \left(AA_{Fi}/AA_{Ei}\right)}\times 100$$

where:

*AA_Fi_* denotes the amount of a certain EAA in the sample, *AA_Ei_* denotes the amount of a certain EAA in the whole egg protein, *AA_Xi_* denotes the amount of a certain EAA recommended by the FAO/WHO model protein, and *i* denotes the number of amino acid species.

The contents of fatty acids were analyzed according to the Chinese standard method GB5009.168-2016 [33].

TPC was evaluated spectrophotometrically using the Folin–Ciocalteu reagent [34]. Gallic acid was used as standard at a concentration of 0.03–2 mg/mL. The reaction system contained 2 mL of Folin–Ciocalteu reagent (1:5 H_2_O), 0.5 mL of H_2_O, 0.5 mL of the sample, and 10 mL of Na_2_CO_3_ (10%) after 3 min. After 30 min, the absorbance was measured at 725 nm against a blank sample using a spectrophotometer (WFZ UV-2802H, Unico Instrument Co., Ltd., China). Values were expressed as milligrams of gallic acid equivalent per gram of dry extract (mg GAE/g). To determine the total flavonoid content (TFC), 0.5 mL of sample was mixed with 0.3 mL of AlCl_3_ (100 g/L), diluted to 2 mL, and measured at a wavelength of 510 nm [35]. Rutin was used as standard at a concentration of 0.03–2 mg/mL. The results were expressed as milligrams of rutin equivalent per gram of dry extract (mg RE/g). As described previously, vanillin-glacial acetic acid reagent was used to measure TSC [36]. Saponin was used as standard at a concentration of 0–500 μg/mL. For this determination, 0.2 mL of sample was mixed with 0.2 mL of vanillin-glacial acetic acid reagent (5%) and 0.8 mL of KMnO_4_, and allowed to react at 70 °C for 15 min before glacial acetic acid (4 mL) was added, and the TSC was immediately measured at a wavelength of 545 nm. Values were calculated as milligrams of saponin per gram of dried material (mg/g).

### 2.6. Analysis of Functional Characteristics

#### 2.6.1. Determination of Antioxidant Properties

Each powder (2.00 g) was mixed evenly with methanol (25 mL) at 25 °C and ultrasonicated for 40 min. Then, the mixture was centrifuged (5000 rpm, 15 min) to obtain the supernatant. The supernatant (0.2 mL) was blended with 1 mL of 0.15 mM DPPH radical solution in methanol, incubated in darkness for 30 min, and analyzed spectrophotometrically at 517 nm [37]. The 2,2′-azino-bis (3-ethylbenzthiazoline-6-sulfonic acid) (ABTS) assays were performed using 0.1 g of ABTS and 0.029 g of K_2_S_2_O_8_ dissolved in phosphate-buffered saline (PBS, pH = 6.6), at a constant volume of 100 mL. ABTS+• was produced via oxidation of ABTS with K_2_S_2_O_8_. After incubation in the dark for 16 h, the mixture was diluted with PBS (pH = 6.6) until the absorbance value was 0.7 ± 0.05 at 734 nm. Each mixture contained the above solution (3.9 mL) and sample (0.1 mL) and was reacted for 5 min, and then absorbance was measured at 734 nm [38]. The calculation formulas are as follows:

$$The\;scanning\;of\;DPPH•\;\left(\%\right)=\left(1-\frac{A_{1}-A_{2}}{A_{3}}\right)\times 100\;The\;scanning\;of\;ABTS+•\;\left(\%\right)=\frac{A_{3}-A_{1}}{A_{3}}\times 100$$

where:

*A*_1_ indicates the absorbance of the sample with the free radical reaction system, *A*_2_ indicates the absorbance of the sample without the free radical reaction system, and *A*_3_ indicates the absorbance of the solvent with the free radical reaction system.

#### 2.6.2. Determination of Hypoglycemic Activities

Glucose adsorption capacity (GAC) [39]: According to the method reported in the literature, 0.10 g of sample was mixed with 5 mL of glucose solution (50, 100, and 200 mmol/L), oscillated at 37 °C for 6 h, and centrifuged at 4000 rpm for 20 min. The supernatant was used for the glucose content determination using the DNS colorimetric method.

$$GAC\;\left(mmol/g\right)=\frac{(c_{0}+c_{1}-c_{2})\times V}{m}$$

where:

*c*_0_ represents the concentration of the glucose solution, *c*_1_ represents the concentration of the sample solution, and *c_2_* represents the concentration of the system after reaction.

For the determination of the glucose dialysis retardation index (GDRI) [39], 0.4 g of sample was mixed with 15 mL of 100 mmol/L glucose solution. Then, the mixture was transferred into a dialysis bag with a molecular weight cutoff of 14,000 and dialyzed against 200 mL of distilled water. From 0 to 150 min, the dialysate was collected every 30 min to determine the glucose content using the DNS colorimetric method.

$$GDRI\;\left(\%\right)=\frac{c_{0}-c}{c_{0}}\times 100$$

where:

*c* is the total glucose diffused from the sample, and *c_0_* is the total glucose diffused from the control.

For the α-amylase activity inhibition ratio (α-AAIR) [39], 0.0250 g of sample was mixed with 75 μL of α-amylase (40 U/mL) and 75 μL of 1% starch solution. The mixture was diluted to 10 mL and incubated at 37 °C for 15 min and bathed in boiling water for 10 min to deactivate the enzymes. After centrifugation at 12,000 rpm for 10 min, the supernatant was used to determine the glucose content.

$$\alpha -\mathrm{AAIR}\;\left(\%\right)=\frac{A_{0}-A_{1}}{A_{0}}\times 100$$

where:

*A*_0_ denotes the glucose concentration of the control, and *A*_1_ denotes the glucose concentration of the sample.

For the α-glucosidase activity inhibition ratio (α-GAIR) [40], α-glucosidase was dissolved in PBS buffer (100 mmol/L, pH = 7.0), 1.0 mg of sample was mixed with 40 μL of 1 U/mL α-glucosidase, and the mixture was incubated at 37 °C for 15 min. Then, 20 μL of 2.5 mmol/L p-nitrophenyl α-D-glucopyranoside was added to the mixture and further incubated at 37 °C for 10 min. Subsequently, 150 μL of 0.2 mol/L Na_2_CO_3_ was added to terminate the reaction, and the absorption was measured at 405 nm.

$$\alpha -\mathrm{GAIR}\;\left(\%\right)=\left(1-\frac{A_{1}-A_{2}}{A_{3}-A_{4}}\right)\times 100$$

where:

*A*_1_ indicates the concentration of the enzyme and sample, *A*_2_ indicates the concentration of the sample and PBS buffer, *A*_3_ indicates the concentration of the enzyme without the sample, and *A*_4_ indicates the concentration of PBS buffer.

#### 2.6.3. Determination of Hypolipidemic Activities

To determine the lipid-binding capacity (LBC) [41], 0.10 g of sample was accurately weighed, mixed with 5 mL of oil, and shaken at 37 °C for 60 min. After centrifugation at 4000 rpm for 20 min, the unbound oil was removed. Soybean oil and lard were chosen as representative unsaturated and saturated fats, respectively.

$$\mathrm{LBC}\left(\%\right)=\frac{m_{1}-m_{0}}{m_{0}}\times 100$$

where:

*m*_0_ is the mass of the sample, and *m*_1_ is the mass of the sample after the adsorption of liquid.

Cholesterol binding capacity (CBC) [42] was determined according to a previously reported method, with slight modifications. A yolk was divided from the egg white and diluted with 9 times the volume of distilled water. Quinoa (0.10 g) was mixed with 4 mL of diluted yolk, and then shaken in a water bath (120 rpm, 37 °C) for 2 h, centrifugated at 4000 rpm for 20 min, and diluted by 10 times. Thereafter, 0.4 mL of supernatant was blended with 1.5 mL of o-phthalaldehyde and 1 mL of H_2_SO_4_, reacted for 10 min in darkness, and measured at 550 nm.

$$\mathrm{CBC}\;\left(\%\right)=\frac{A_{0}-A_{1}}{A_{0}}\times 100$$

where:

*A*_0_ is the cholesterol concentration of the control, and *A*_1_ is the cholesterol concentration of the sample.

To determine the bile salt-binding capacity (BBC) [43], four cholate salts (sodium cholate, sodium taurocholate, sodium deoxycholate, and sodium glycocholate) were used. According to the method reported in the literature, the samples (0.0250 g) were added with 0.01 mol/L HCl (0.25 mL) and oscillated at 37 °C for 1 h, simulating gastric digestion in vitro. The reaction mixture, the pH of which was adjusted to 7.0, was added to 4 mL of 2 g/L bile solution, dissolved in 0.01 mol/L phosphate buffer at pH 7.0. It was shaken at 37 °C for 20 min to simulate intestinal digestion in vitro, and centrifuged at 8000 rpm for 20 min. Then, 0.3 mL of supernatant was blended with 3 mL of H_2_SO_4_ (45%, *v*/*v*) and subjected to a water bath at 70 °C for 20 min and an ice water bath for 5 min. The concentration was measured at 387 nm.

$$\mathrm{BBC}\;\left(\%\right)=\frac{A_{0}-A_{1}}{A_{0}}\times 100$$

where:

*A*_0_ is the initial concentration of the bile salt, and *A*_1_ is the concentration of the bile salt after adsorption.

For determining the pancreatic lipase activity inhibition ratio (PAIR) [44], 0.050 g of sample, 0.1 mL of porcine pancreatic lipase (60 U/mL), 1 mL of soybean oil, and 5 mL of phosphate buffer (0.1 mol/L, pH 7.0) were thoroughly mixed together and oscillated at 37 °C for 1 h. The mixture was bathed in boiling water for 10 min to terminate the reaction. Then, NaOH standard solution (0.01 mol/L) was used for titration with phenolphthalein as an indicator.

$$\mathrm{PAIR}\;\left(\%\right)=\frac{V_{0}-V_{1}}{V_{0}}\times 100$$

where:

*V*_0_ is the titration volume of NaOH without adsorption, and *V*_1_ is the titration volume of NaOH with adsorption.

### 2.7. Statistical Analysis

The data reported in this study are expressed as the mean ± S.D. One-way ANOVA, performed with SPSS version 25 (SPSS Institute, Chicago, IL, USA), was used to identify significant differences between the sample means. Values of *p* < 0.05 and *p* < 0.01 indicate significant and extremely significant differences, respectively.

## 3. Results and Discussion

### 3.1. Effects of Ultrasonic Treatment on the GABA Accumulation of Quinoa during Germination

**Figure 2:**
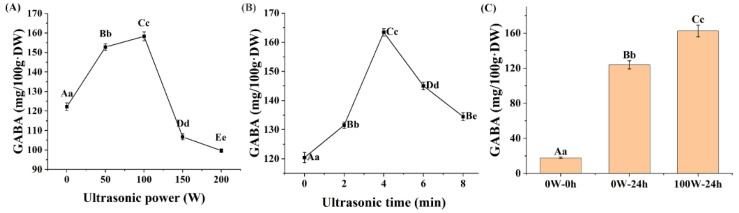
Effects of ultrasonic treatment on the γ-aminobutyric acid (GABA) contents of quinoa sprouts ^1^ ((**A**) effects of ultrasonic power on the GABA contents of quinoa sprouts ^2^; (**B**) effects of ultrasonic time on the GABA contents of quinoa sprouts ^3^; and (**C**) the GABA contents at 0 W–0 h, 0 W–24 h, and 100 W–24 h ^4^). ^1^ Different lowercase and capital letters indicate significant and extremely significant differences, at *p* < 0.05 and *p* < 0.01, respectively. ^2^ Quinoa was treated with ultrasound for 4 min. ^3^ Quinoa was treated with ultrasound at 100 W. ^4^ 0 W–0 h, quinoa plants without ultrasonic stimulation and germination; 0 W–24 h, quinoa plants with 24 h germination; 100 W–24 h, ultrasound-treated (100 W, 4 min) quinoa plants with 24 h germination.

GABA is widely distributed in plant species at low levels [15] and it accumulates under stress conditions as a stress response factor [16]. Ultrasound is an environmentally friendly and non-thermal stress method. As shown in Figure 2A,B, the GABA contents reached a peak value under 100 W and 4 min of ultrasonic treatment after germination, and then decreased. Compared with quinoa without ultrasonic stimulation and germination (17.75 ± 0.83 mg/100 g·DW), and quinoa with 24 h germination without ultrasonic stimulation (123.85 ± 4.66 mg/100 g·DW), the GABA content of 24 h germinated quinoa treated with 100 W ultrasonication for 4 min (162.47 ± 06.69 mg/100 g·DW) increased by 815.3% and 31.2%, respectively (Figure 2C). Studies on red rice [18], soybean [22], maize [45], oat [19], and whole-grain brown rice [20] showed that GABA contents could be enriched via ultrasound treatment, to 41.58 mg/100 g·DW–338.38 mg/100 g·DW, which represents a range of 5.36–69.2%. Furthermore, the application of ultrasound stress after germination could generate more GABA in quinoa per unit of time than those generated in the above cereals.

**Figure 3:**
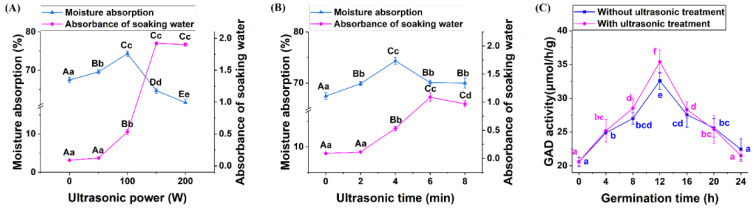
Effects of ultrasonic treatment on the moisture absorption, turbidity of soaking water, and glutamate decarboxylase (GAD) activity of quinoa sprouts ^1^ ((**A**) effects of ultrasonic power on the moisture absorption and turbidity of soaking water of quinoa ^2^; (**B**) effects of ultrasonic time on the moisture absorption and turbidity of soaking water of quinoa ^3^; and (**C**) effects of ultrasonic treatment at 100 W for 4 min on the GAD activity of quinoa sprouts ^4^). ^1^ Different lowercase and capital letters indicate significant and extremely significant differences, at *p* < 0.05 and *p* < 0.01, respectively. ^2^ Quinoa was treated with ultrasound for 4 min. ^3^ Quinoa was treated with ultrasound at 100 W. ^4^ Quinoa was treated with ultrasound for 4 min at 100 W.

During the ultrasonic process in an aqueous medium, mechanical and chemical effects on seeds are induced by the collapse of cavitation bubbles, which are produced through a series of compression and rarefaction processes [46]. As observed in Arabidopsis, low-frequency short-duration ultrasonic treatment promoted water uptake and oxygen supply because ultrasonic cavitation altered the permeability of the cell membrane [47]. Correspondingly, a 10.21% increase in water absorption after ultrasonic treatment (100 W, 4 min) was observed (Figure 3A,B), which may promote the activities of enzymes required for germination. As shown in Figure 3C, at 12 h of germination, ultrasonic stress caused a significant increase in GAD activity, which irreversibly converted glutamate to GABA [48]. However, with increasing sonication time or power, physical and chemical damage occurs, owing to the very rapid local changes in temperature and pressure caused by ultrasound waves, which can produce fatal effects on seeds soaked in a water bath [49]. The results showed that the turbidity of the quinoa soaking water increased and the water absorption decreased significantly when the ultrasonic conditions exceeded 100 W or 4 min, indicating that the cell membrane and seed coat, as physical barriers for quinoa, were probably damaged. Thus, proper ultrasonic stress may have enhanced the GAD activity of quinoa seeds by promoting cell wall permeability and water absorption, which, in turn, facilitated GABA accumulation.

The previous experimental results indicated that the GABA content of quinoa sprouts peaked at 24 h of germination without ultrasonic stress. Further experiments revealed that ultrasonic treatment at 100 W for 4 min at the pre-germination stage was the optimal enrichment condition for GABA. Therefore, the samples were divided into three groups: 0 W–0 h, quinoa plants without ultrasonic stimulation and germination; 0 W–24 h, quinoa plants with 24 h germination; 100 W–24 h, ultrasound-treated (100 W, 4 min) quinoa plants with 24 h germination. The three treatments of quinoa (0 W–0 h, 0 W–24 h, and 100 W–24 h) were used for subsequent experiments.

### 3.2. Effects of Ultrasonic Treatment on the Nutritional Quality of Quinoa during Germination

#### 3.2.1. Effects of Ultrasonic Treatment on the Basic Nutritional Components in Quinoa during Germination

**Figure 4:**
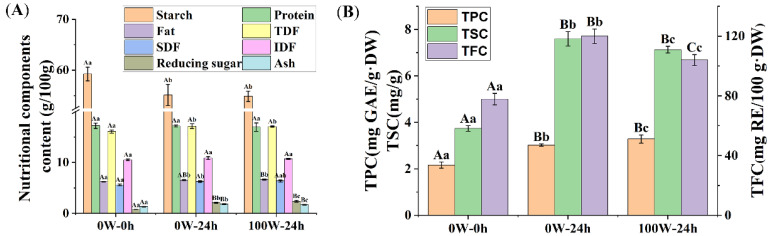
Effects of ultrasonic treatment on the basic nutritional components (**A**) and phytochemical composition (**B**) in sprouts ^1^.^1^ For the 0 W–0 h quinoa and the 0 W–24 h, and 100 W–24 h treatments, different lowercase and capital letters were used to indicate significant and extremely significant differences, at *p* < 0.05 and *p* < 0.01, respectively.

The changes in the basic nutritional components, such as the starch, protein, fat, total dietary fiber (TDF), soluble dietary fiber (SDF), insoluble dietary fiber (IDF), reducing sugar, and ash contents of quinoa are shown in Figure 4A. Compared with the 0 W–0 h quinoa, the starch contents of the 0 W–24 h quinoa decreased by 6.99%, while the fat, TDF, SDF, IDF, reducing sugar, and ash contents increased by 4.92%, 6.52%, 12.34%, 3.44%, 171.98%, and 38.53%, respectively (*p* < 0.05). Compared with the 0 W–24 h quinoa, the ash contents of the 100 W–24 h quinoa decreased by 8.14%, while the reducing sugar contents increased by 13.01% (*p* < 0.05). Significant differences were not observed in the protein content of quinoa samples among the 0 W–0 h quinoa and the 0 W–24 h, and 100 W–24 h treatments. This suggests that ultrasonic stimulation prior to sprouting can accelerate starch decomposition, as observed in whole-grain brown rice [20]. This phenomenon could be related to the more active enzymatic hydrolysis process under ultrasonic treatment, which disrupts starch–lipid and starch–protein compounds and promotes starch hydrolysis, thus leading to increased sensitivity of starch to amylase; however, amylase activity could be impaired with ultrasound treatment, depending on the treatment conditions [50,51]. In addition, proteolysis caused by germination does not significantly change the total protein content and was more severe after at least two days of germination [52]. In this experiment, ultrasound treatment and germination did not significantly reduce protein content, possibly because the germination time was only 24 h.

**Figure 5:**
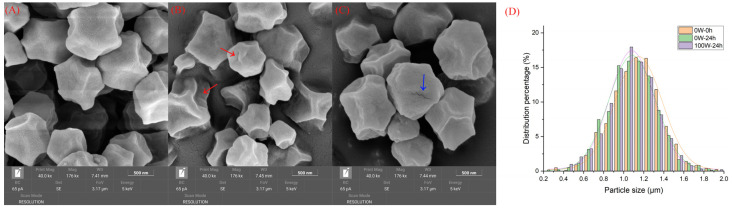
Effects of ultrasonic treatment on the microstructures ^1^ ((**A**) 0 W–0 h; (**B**) 0 W–24 h; and (**C**) 100 W–24 h) and particle size distributions (**D**) of starch samples extracted from quinoa samples under different treatments during germination. The image magnification was approximately 40 k× for whole granules. ^1^ The red and blue arrows were used to point to crimples and fissures, respectively.

To further illustrate the effects of ultrasound treatment on quinoa starch granule morphology and granule size distribution, the microstructure of the starch was characterized using SEM. Quinoa starch exhibited granular polygons, and the particle surface appeared rough (Figure 5A–C), which is consistent with previous reports [53]. On the surfaces of the quinoa starch particles, germination for 24 h increased crimples, and ultrasound stress caused fissures that increased the exposure of starch to enzymes and ultimately promoted the hydrolysis of starch and the biosynthesis of reducing sugar. Previous studies have shown that more pinholes and evident curls appeared on the surface of sprouting quinoa starch particles, compared with the surface of native starch [54]. Meanwhile, germination for 24 h shifted the bell-shaped particle size distribution to the left. This changing pattern was enhanced through ultrasound stimulation (Figure 5D). The average particle size of quinoa starch ranged from 1.0809 ± 0.0044 μm (100 W–24 h) to 1.1483 ± 0.0066 μm (0 W–0 h), indicating that the quinoa starch granule sizes significantly decreased, by 5.87%, under ultrasound and germination. These results were consistent with the observation of the effect of ultrasonic pre-treatment on whole rice starch during germination, which was attributed to ultrasound promoting the degradation of starch particles in reaction to enzymatic attack [20].

**Figure 6:**
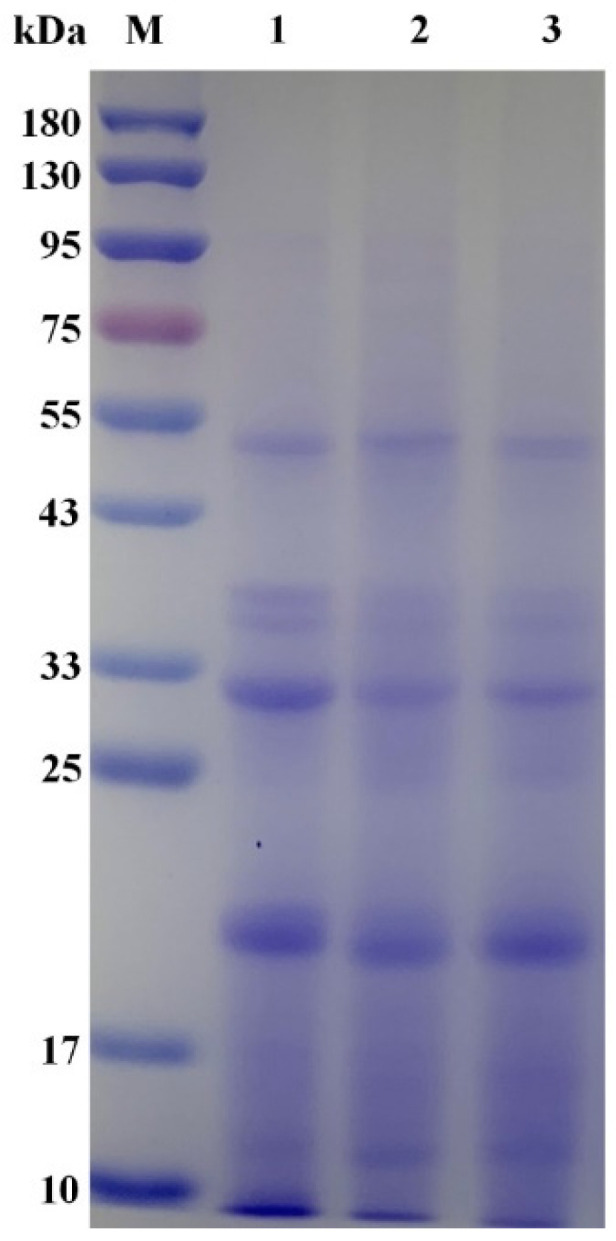
Effects of ultrasonic treatment and germination on the protein profiles of quinoa. (M, standard protein marker; lane 1, 0 W–0 h; lane 2, 0 W–24 h; lane 3, 100 W–24 h).

Differences in the protein patterns among quinoa samples were analyzed using SDS-PAGE, as presented in Figure 6. According to previous studies, quinoa proteins are composed of 11S-globulin (containing peptides with molecular weights of 22–23 kDa and 32–39 kDa) and 2S-protein (with a molecular weight of 9 kDa) [29]. The electrophoretic protein patterns on the gels were relatively constant. However, some protein bands from the 0 W–24 h and 100 W–24 h treatments, on the electrophoresis gel, showed lower intensities compared with those from the 0 W–0 h quinoa. Proteolysis was more pronounced for proteins ranging from 33 to 43 kDa. Germination and ultrasound treatment had effects on the peptide chains of the proteins, as indicated by changes in the intensities of some protein bands. Similar tendencies have been shown in soybean and oat [22,55].

The changes in the amino acid contents of quinoa are shown in Table 1. Notably, the levels of lysine in all quinoa samples (4.19–4.43 g/100 g protein) were higher than those in barley (2.32–2.82 g/100 g protein), wheat (1.09–1.51 g/100 g protein), and rye (1.97–3.42 g/100 g protein) [56]. Meanwhile, ultrasound treatment was only beneficial for accumulating total acid amino acids, not total basic amino acids. Considering the proteins of the 0 W–0 h quinoa, leucine, glutamate, and isoleucine were the three main amino acids, while, among the proteins of the 0 W–24 h and 100 W–24 h treatments, leucine, glutamate, and methionine were the three main amino acids, accounting for 24.41–25.06% of total amino acids. Germination and ultrasound treatment promoted the accumulation of aspartate, glutamate, alanine, and proline. Studies have shown that proline accumulation is related to abiotic stress tolerance [57]. Alanine, which promoted the cellular energy metabolism of germinated rice, showed similar trends as GABA under ultrasonic treatment [18]. Pyruvate, α-ketoglutaric acid, and oxaloacetic acid, produced during the aerobic decomposition of sugar, can be aminated and converted into alanine, glutamate, and aspartate, respectively. To resist the oxidative stress caused by abiotic stress, GABA can be synthesized from glutamate and arginine through the GABA shunt and polyamine degradation pathways, respectively. This finding shows that the abiotic stress caused by ultrasound treatment can be alleviated by improving carbon metabolism and amino acid biosynthesis during the germination of quinoa. Meanwhile, germination increased the contents of valine and methionine and decreased the contents of isoleucine and leucine. In a study on mung bean, germination increased the contents of valine and methionine [31]. As shown in Table 1, ultrasound treatment also enhanced the accumulation of phenylalanine and the consumption of valine, methionine, isoleucine, and arginine. Polyamines are derived from arginine [58], which is further synthesized into GABA. As shown in Table 2, germination and sonication had essentially no effect on the nutritional value of essential amino acids, but it is noteworthy that germination and sonication slightly increased the amino acid score (AAS) and chemical score (CS) values for lysine.

Quantitative analysis of the composition of 37 fatty acids in quinoa was conducted using gas chromatography. As shown in Table 3, 14 fatty acids were detected, and they were all long-chain fatty acids. In all quinoa samples, linoleate (52.90–53.77%), oleate (20.06–21.46%), palmitate (9.67–10.00%), and alpha linoleate (8.74–9.34%) were the main fatty acids. Moreover, n6/n3 polyunsaturated fatty acids (PUFAs) were improved by 4.85% and 6.79% via germination and ultrasound treatment, respectively, and these values are closer to the recommended daily intake in a healthy diet (4:1). The n6/n3 PUFA ratio of the 100 W–24 h quinoa (5.48/1) was lower than those of olive oil (13.4/1), corn oil (52/1) [59], soybean oil (7.4/1), and Spanish experimental quinoa (9/1) [60], and better than the average ratios in both Western diets (15.0/1–16.7/1) and Chinese diets (20/1–30/1) [61]. An imbalance in n6/n3 PUFAs is associated with inflammatory diseases, including diabetes, cardiovascular diseases, and cancer [61].

#### 3.2.2. Effects of Ultrasonic Treatment on the Phytochemical Composition of Quinoa during Germination

As shown in Figure 4B, the TPC, TFC, and TSC of the 0 W–24 h quinoa were 39.76%, 27.43%, and 103.21% higher than those of the 0 W–0 h quinoa (*p* < 0.01). The same trends for the TPC and TFC were found in mung bean seeds [31], amaranth flour [62], fava bean [34], and soybean [63]. In fava bean, the levels of saponins increased by germination [34]. However, another study on quinoa revealed that the saponin content, of 0.860 mg/100 g, decreased to 0.086 mg/100 g after 48 h of germination [64]. In addition to the controversy regarding the effect of germination on saponin content, various studies have found that the TPC and TFC were promoted by germination, which is consistent with the experimental results in this study. However, changes in the TPC, TFC, and TSC values were relatively complex under abiotic stress. Compared with the 0 W–24 h quinoa, the TPC of the 100 W–24 h quinoa increased by 8.7%, whereas the TFC and TSC of the 100 W–24 h quinoa decreased by 13.32% and 6.18%, respectively (Figure 2B). In oats, ultrasound treatment increased the TPC level [19]. Meanwhile, the TSC was reduced in tomato and cucumber under salinity stress, whereas the TPC increased [65]. A study on *Haloxylon stocksii*, a moderately salt-tolerant plant, showed that its TFC and TSC levels decreased under saline conditions [66]. The above research results are consistent with the experimental results in this study. Plants under abiotic stress (including ultrasonic stress) regulate the biosynthesis and consumption of polyphenols, flavonoids, and saponins in response to oxidative stress, which involve the removal of reactive oxygen species and the phenylpropanoid pathway, as well as other biosynthetic pathways. Among them, saponins that protect plants against oxidative stress are attracting increasing attention.

### 3.3. Effects of Ultrasonic Treatment on the Functional Characteristics of Quinoa during Germination

To explore the effects of ultrasound stress on the functional properties of quinoa, we determined the antioxidant, hypoglycemic, and hypolipidemic capacities of quinoa.

#### 3.3.1. Effects of Ultrasonic Treatment on the Antioxidant Properties of Quinoa during Germination

**Figure 7:**
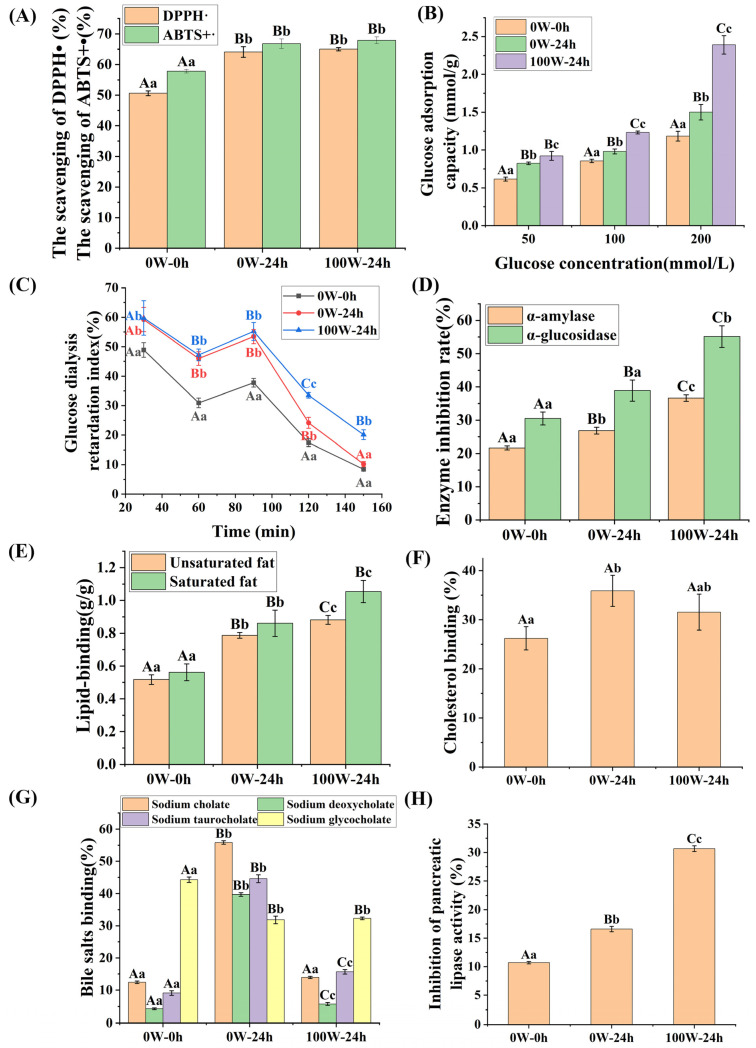
Effects of ultrasonic treatment on the functional characteristics of quinoa during germination ^1^ ((**A**) antioxidant property; (**B**) glucose adsorption capacity; (**C**) glucose dialysis retardation index; (**D**) α-amylase and α-glucosidase activity inhibition ration; (**E**) lipid-binding capacity; (**F**) cholesterol-binding capacity; (**G**) bile salt-binding capacity; (**H**) inhibition of pancreatic lipase activity). ^1^ Among the 0 W–0 h quinoa and the 0 W–24 h, and 100 W–24 h treatments, different lowercase and capital letters indicate significant and extremely significant differences, at *p* < 0.05 and *p* < 0.01, respectively.

The scavenging of DPPH• and ABTS+• among the treatments followed a descending order of 100 W–24 h ≥ 0 W–24 h > 0 W–0 h, 100 W24 h ≥ 0 W–24 h > 0 W–0 h (Figure 7A). Free radical scavenging was markedly enhanced by germination, which was further promoted by ultrasonic treatment, with no statistical difference. These data indicate that the scavenging of DPPH• and ABTS+• were positively associated with the TPC. Research on peanuts has shown that the TPC and TFC values, as well as the antioxidant activity, are significantly promoted by germination, and the antioxidant activity of germinated peanut extract is closely related to its TPC and TFC [67]. Furthermore, saponins that defend against oxidative stress have attracted increasing attention. Alfalfa saponins have been identified as potential inartificial antioxidants, owing to their remarkable antioxidant activity [68], and notoginsenoside R1 can alleviate neuronal injury by restraining the production of reactive oxygen [69]. Compared with ungerminated amaranth flours, germinated samples exhibited an increase in antioxidant activity, by 54.3% [62]. Various studies have indicated that the antioxidant activities of wheat [70], barley [71], brown rice [72], and oats [73] increases by 20–190% after germination (15–28 °C, 2–5 d). Sprouting generally promotes antioxidant activity in cereals, which is consistent with this study. However, the effects of abiotic stress on antioxidant activity are relatively complex. In oats, ultrasound treatment increased antioxidant activity [19], whereas, in tomato and cucumber plants, salinity stress reduced antioxidant activity [65].

#### 3.3.2. Effects of Ultrasonic Treatment on the Hypoglycemic Activity of Quinoa during Germination

The hypoglycemic effects of quinoa are shown in Figure 7B–D. Figure 7B shows that the GAC of the 0 W–0 h quinoa and the 0 W–24 h, and 100 W–24 h treatments increased, from 0.62 mmol/g to 2.39 mmol/g, successively. Figure 7C shows that the 100 W–24 h treatment generated the highest GDRI, followed by the 0 W–24 h treatment, with both rates exceeding that of the 0 W–0 h treatment. GDRI is an effective index in vitro and is frequently used to calculate the delay of glucose absorption in the gastrointestinal tract. GAC and GDRI might be related to the content and structure of DF and the amounts of functional groups exposed to glucose in quinoa. These groups (especially the phenolic group), with outstanding affinity to glucose, can effectively inhibit the diffusion of glucose [74]. High concentrations of SDF can delay the diffusion of glucose by enhancing the binding of glucose molecules to the fiber network [75]. For DF, ultrasound treatment and enzymolysis resulted in a honeycomb network structure, which increased the contact area with glucose, leading to relatively high GAC and GDRI values [39,76]. These results indicated that ultrasound treatment and germination enhanced the GAC of quinoa, which might benefit the delay of glucose absorption in the gastrointestinal tract and inhibit the rise of postprandial blood glucose. The strategy of controlling hyperglycemia via inhibition of α-amylase and α-glucosidase, associated with carbohydrate digestion, is considered as a viable prophylactic treatment [77]. Pairwise comparisons of three quinoa samples showed that germination increased α-AAIR and α-GAIR by 23.64% and 27.53%, respectively, and ultrasound treatment further enhanced α-AAIR and α-GAIR by 36.62% and 41.77%, respectively (Figure 7D). The DF prevents the reaction of the enzyme and substrate, which inhibits the catalytic efficiency of starch hydrolysis [78], while phenolic substances compete with the substrate for the active binding sites of α-amylase and α-glucosidase [79]. The results indicated that quinoa flours subjected to ultrasound treatment and germination present decreased generation of glucose and sorption of glucose in the gastrointestinal tract, thus leading to a lower postprandial blood glucose.

#### 3.3.3. Effects of Ultrasonic Treatment on the Hypolipidemic Activity of Quinoa Plants during Germination

The results of the hypolipidemic effects of quinoa are shown in Figure 7E–H. As shown in Figure 7E, the 100 W–24 h treatment generated the highest LBC, followed by the 0 W–24 h treatment, and both values exceeded those of the 0 W–0 h quinoa. Moreover, the holding capacities of saturated fat in quinoa were better than those of unsaturated fat, which are important indicators for predicting weight loss. Quinoa samples are rich in polysaccharides and dietary fiber, and their honeycomb network structure might be enhanced by ultrasound treatment and germination [76,80]. The above evidence indicated that ultrasound treatment and germination might increase the contact areas between the quinoa flours and liquid and cholesterol, forming macromolecules in the gastrointestinal tract to prevent them from being absorbed by the human body [81].

According to Figure 7F, germination increased the CBC by 36.93% (*p* < 0.05), while ultrasound treatment had no significant effect on it (*p* > 0.05). Cholesterol is a principal factor underlying cardiovascular disease. Polysaccharides and DF, with a honeycomb network structure, could also enhance the cholesterol adsorption capacity. Cholic, deoxycholic, glycocholic, and taurocholic acids are the major bile acids in the human body, and over 90% exist in a bound state (especially sodium salts). Bile acids are adsorbed in the intestine and excreted to the outside of the body, which can promote the conversion of cholesterol into free bile acid in the liver. In this manner, the cholesterol and lipid levels in the body could be reduced [42]. As shown in Figure 7G, the comparison between the 0 W–24 h treatment and the 0 W–0 h quinoa showed that germination increased the binding capacity of sodium cholate, sodium deoxycholate, and sodium taurocholate by 349.0%, 821.4%, and 391.4%, respectively (*p* < 0.01). The comparison between the 100 W–24 h and 0 W–24 h treatments showed that ultrasound treatment reduced the binding capacity of sodium cholate, sodium deoxycholate, and sodium taurocholate by 74.99%, 85.52%, and 64.85%, respectively (*p* < 0.01), and these values were still higher than those of the 0 W–0 h quinoa. The binding capacities of sodium glycocholate of the 0 W–0 h quinoa and the 0 W–24 h, and 100 W–24 h treatments were 44.27 ± 0.85%, 31.83 ± 1.16%, and 32.29 ± 0.42%. Ultrasound treatment and germination have different effects on the binding capacities of different bile acids, which may be related to the characteristics of each bile acid. This trend is similar to previous research results [43]. The adsorption of bile acids, hydrophobic substances, depends on hydrophobic and electrostatic interactions. However, ultrasound treatment exposed a large number of hydrophilic groups in protein and dietary fiber [80,82], resulting in a significant decline in the BBC.

Figure 7H shows that germination and ultrasound treatment increased the PAIR by 55.23% and 84.64% (*p* < 0.01), respectively. Germination significantly increased the TPC and TSC values in quinoa, which have been reported to inhibit pancreatic lipase [83]. Ultrasound treatment made the structure of DF more loose, increased the binding with pancreatic lipase, reduced the contact between pancreatic lipase and substrate, and thus increased the PAIR [84]. In summary, germination can theoretically improve hypolipidemic activity, while ultrasound treatment affects hypolipidemic activity, based on a variety of factors that can be verified in vivo.

## 4. Conclusions

In this study, we investigated the changes in GABA content and the nutritional and functional attributes of quinoa under pre-germination ultrasound stress. Ultrasonic conditions substantially promoted GABA content, compared with that recorded in untreated quinoa, and enhanced the enrichment of some secondary metabolites, such as TPC, without reducing basic nutritional qualities. Meanwhile, ultrasonic stress and germination increased crimples and fissures on the surface of quinoa starch particles, and it also promoted the hydrolysis of starch and the biosynthesis of reducing sugar. Ultrasound stress optimized the composition of fatty acids, including n3 PUFAs (% of TFAs) and the n6/n3 PUFA ratio. Furthermore, the antioxidant, hypolipidemic, and hypoglycemic capacities of quinoa in vitro could be improved through ultrasonic-stimulated pre-germination. Our results provide data for further research on the effects of ultrasonic stress on grains and theoretical support for the intensive processing and development of functional whole-grain foods based on quinoa. Further mechanistic studies and long-term animal studies are warranted to determine the principles of ultrasonic stress and the functional properties of ultrasound-stressed germinated quinoa.

## Figures and Tables

**Table 1:** Effects of ultrasonic treatment on the amino acid contents of quinoa during germination ^1^.

Amino Acid (g/100g Protein)	0 W–0 h	0 W–24 h	100 W–24 h
Threonine	2.93 ± 0.02 ^Aa^	2.92 ± 0.01 ^Aa^	2.91 ± 0.04 ^Aa^
Valine	5.22 ± 0.02 ^Aa^	6.29 ± 0.04 ^Bb^	5.54 ± 0.02 ^Cc^
Methionine	6.38 ± 0.01 ^Aa^	7.50 ± 0.03 ^Bb^	7.42 ± 0.03 ^Cc^
Isoleucine	7.64 ± 0.02 ^Aa^	7.18 ± 0.04 ^Bb^	6.81 ± 0.02 ^Cc^
Leucine	9.36 ± 0.04 ^Aa^	8.80 ± 0.02 ^Bb^	8.75 ± 0.05 ^Bb^
Phenylalanine	2.81 ± 0.02 ^Aa^	2.84 ± 0.01 ^Aa^	2.97 ± 0.02 ^Bb^
Lysine	4.19 ± 0.01 ^Aa^	4.43 ± 0.02 ^Bb^	4.34 ± 0.03 ^Cc^
Histidine	2.36 ± 0.01 ^Aa^	2.32 ± 0.01 ^Bb^	2.36 ± 0.02 ^Aa^
Arginine	5.23 ± 0.01 ^Aa^	5.43 ± 0.02 ^Bb^	5.36 ± 0.03 ^Bc^
Aspartate	6.11 ± 0.01 ^Aa^	6.48 ± 0.03 ^Bb^	6.69 ± 0.03 ^Cc^
Serine	2.91 ± 0.01 ^Aa^	3.15 ± 0.04 ^Bb^	3.10 ± 0.03 ^Bb^
Glutamate	8.06 ± 0.06 ^Aa^	8.31 ± 0.03 ^Bb^	8.24 ± 0.04 ^Bb^
Glycine	3.36 ± 0.01 ^Aa^	3.38 ± 0.01 ^Aa^	3.47 ± 0.02 ^Bb^
Alanine	2.61 ± 0.01 ^Aa^	3.03 ± 0.02 ^Bb^	3.38 ± 0.03 ^Cc^
Cysteine	0.88 ± 0.01 ^Aa^	0.87 ± 0.01 ^Aa^	0.87 ± 0.02 ^Aa^
Tyrosine	3.07 ± 0.02 ^Aa^	3.12 ± 0.02 ^Ab^	2.94 ± 0.01 ^Bc^
Proline	4.77 ± 0.01 ^Aa^	5.72 ± 0.09 ^Bb^	6.26 ± 0.07 ^Cc^
Total amino acids	77.89 ± 0.06 ^Aa^	81.78 ± 0.17 ^Bb^	81.40 ± 0.04 ^Cc^
Total hydrophobic amino acids	38.80 ± 0.04 ^Aa^	41.37 ± 0.13 ^Bb^	41.13 ± 0.05 ^Cc^
Total hydrophilic amino acids	39.10 ± 0.04 ^Aa^	40.41 ± 0.04 ^Bb^	40.27 ± 0.07 ^Bc^
Total acid amino acids	14.16 ± 0.05 ^Aa^	14.79 ± 0.02 ^Bb^	14.92 ± 0.01 ^Cc^
Total basic amino acids	34.32 ± 2.87 ^Aa^	35.56 ± 3.11 ^Aa^	35.19 ± 2.98 ^Aa^
Total aromatic amino acids	5.88 ± 0.04 ^Aa^	5.96 ± 0.02 ^Bb^	5.91 ± 0.01 ^ABac^
Total essential amino acids	38.53 ± 0.06 ^Aa^	39.97 ± 0.11 ^Bb^	38.74 ± 0.03 ^Cc^
Total half essential amino acids	7.59 ± 0.01 ^Aa^	7.75 ± 0.03 ^Bb^	7.72 ± 0.05 ^Bb^

^1^ In the same row, different lowercase and capital letters indicate significant and extremely significant differences, at *p* < 0.05 and *p* < 0.01, respectively. The data reported in this chart are expressed as the mean ± S.D (*n* = 3).

**Table 2:** Effects of ultrasonic treatment on the nutritional values of quinoa during germination ^1^.

Evaluation Criterion	Amino Acid	0 W–0 h	0 W–24 h	100 W–24 h
AAS	Isoleucine	2.73	2.56	2.43
	Leucine	1.42	1.33	1.32
	Lysine	0.72	0.76	0.75
	Methionine + Cysteine	2.90	3.35	3.31
	Phenylalanine + Tyrosine	0.93	0.95	0.93
	Valine	1.54	1.85	1.63
	Threonine	0.84	0.83	0.83
CS	Isoleucine	1.41	1.33	1.26
	Leucine	1.09	1.02	1.02
	Lysine	0.60	0.63	0.62
	Methionine + Cysteine	1.27	1.47	1.45
	Phenylalanine + Tyrosine	0.63	0.64	0.64
	Valine	0.79	0.95	0.84
	Threonine	0.62	0.62	0.62
EAAI		86.64	90.05	87.16

^1^ AAS: amino acid score; CS: chemical score; EAAI: essential amino acid index.

**Table 3:** Effects of ultrasonic treatment on the fatty acid composition of quinoa during germination ^1,2^.

Fatty Acid	Abbreviation	0 W–0 h	0 W–24 h	100 W–24 h
SFAs (g/100 g)				
Myristate	C14:0	0.0109 ± 0.0001 ^Aa^	0.0121 ± 0.0001 ^Bb^	0.0126 ± 0.0000 ^Cc^
Pentadecanoate	C15:0	0.0037 ± 0.0000 ^Aa^	0.0044 ± 0.0000 ^Bb^	0.0044 ± 0.0000 ^Bb^
Palmitate	C16:0	0.5545 ± 0.0035 ^Aa^	0.5959 ± 0.0013 ^Bb^	0.6032 ± 0.0010 ^Cc^
Stearate	C18:0	0.0276 ± 0.0011 ^Aa^	0.0336 ± 0.0002 ^Bb^	0.0327 ± 0.0003 ^Bb^
Arachidate	C20:0	0.0263 ± 0.0003 ^Aa^	0.0279 ± 0.0003 ^Bb^	0.0285 ± 0.0000 ^Bc^
Behenate	C22:0	0.0358 ± 0.0001 ^Aa^	0.0412 ± 0.0003 ^Bb^	0.0427 ± 0.0002 ^Cc^
Tricosanoate	C23:0	0.1294 ± 0.0007 ^Aa^	0.1078 ± 0.0015 ^Bb^	0.0959 ± 0.0001 ^Cc^
Lignocerate	C24:0	0.0205 ± 0.0004 ^Aa^	0.0258 ± 0.0004 ^Bb^	0.0277 ± 0.0007 ^Cc^
MUFAs (g/100 g)				
Oleate	C18:1N9C	1.2299 ± 0.0073 ^Aa^	1.2057 ± 0.0092 ^Bb^	1.2309 ± 0.0024 ^Aa^
11-eicosenoate	C20:1	0.0772 ± 0.0007 ^Aa^	0.0789 ± 0.0010 ^ABb^	0.0799 ± 0.0007 ^Bb^
Nervonoate	C24:1	0.0100 ± 0.0001 ^Aa^	0.0108 ± 0.0001 ^Bb^	0.0109 ± 0.0002 ^Bb^
PUFAs (g/100 g)				
Linoleate	C18:2N6	3.0319 ± 0.0200 ^Aa^	3.2308 ± 0.0287 ^Bb^	3.2291 ± 0.0056 ^Bb^
Alpha linolenate	C18:3N3	0.5012 ± 0.0033 ^Aa^	0.5610 ± 0.0065 ^Bb^	0.5605 ± 0.0019 ^Bb^
Arachidonate	C20:4N6	0.0722 ± 0.0005 ^Aa^	0.0732 ± 0.0007 ^Ab^	0.0750 ± 0.0001 ^Bc^
TFAs (g/100 g)		5.7311 ± 0.0378 ^Aa^	6.0094 ± 0.0498 ^Bb^	6.0341 ± 0.0107 ^Bb^
SFAs (% of TFAs)		14.11 ± 0.02 ^Aab^	14.13 ± 0.05 ^Aa^	14.05 ± 0.01 ^Ab^
MUFAs (% of TFAs)		22.98 ± 0.01 ^Aa^	21.56 ± 0.01 ^Bb^	21.90 ± 0.01 ^Cc^
PUFAs (% of TFAs)		62.90 ± 0.01 ^Aa^	64.32 ± 0.06 ^Bb^	64.05 ± 0.02 ^Cc^
UFAs (% of TFAs)		85.89 ± 0.02 ^Aab^	85.88 ± 0.06 ^Aa^	85.95 ± 0.02 ^Ab^
n3 PUFAs (% of TFAs)		8.74 ± 0.00 ^Aa^	9.34 ± 0.03 ^Bb^	10.00 ± 0.00 ^Cc^
n6 PUFAs (% of TFAs)		54.16 ± 0.01 ^Aa^	54.98 ± 0.03 ^Bb^	54.76 ± 0.01 ^Cc^
n6/n3 PUFAs		6.19 ± 0.00 ^Aa^	5.89 ± 0.01 ^Bb^	5.48 ± 0.00 ^Cc^

^1^ In the same row, different lowercase and capital letters were used to indicate significant and extremely significant differences, at *p* < 0.05 and *p* < 0.01, respectively. The data reported in this chart are expressed as the mean ± S.D (*n* = 3). ^2^ SFA: saturated fatty acid; MUFA: monounsaturated fatty acid; PUFA: polyunsaturated fatty acid; TFA: total fatty acid.

## Data Availability

Data is contained within the article.
